# Experimental test of the no-go theorem for continuous *ψ*-epistemic models

**DOI:** 10.1038/srep26519

**Published:** 2016-05-31

**Authors:** Kai-Yu Liao, Xin-Ding Zhang, Guang-Zhou Guo, Bao-Quan Ai, Hui Yan, Shi-Liang Zhu

**Affiliations:** 1 Guangdong Provincial Key Laboratory of Quantum Engineering and Quantum Materials, School of Physics and Telecommunication Engineering, South China Normal University, Guangzhou 510006, China; 2 National Laboratory of Solid State Microstructures, School of Physics, Nanjing University, Nanjing 210093, China

## Abstract

Quantum states are the key mathematical objects in quantum theory; however, there is still much debate concerning what a quantum state truly represents. One such century-old debate is whether a quantum state is ontic or epistemic. Recently, a no-go theorem was proposed, stating that the continuous *ψ*-epistemic models cannot reproduce the measurement statistic of quantum states. Here we experimentally test this theorem with high-dimensional single photon quantum states without additional assumptions except for the fair-sampling assumption. Our experimental results reproduce the prediction of quantum theory and support the no-go theorem.

Debates over fundamental concepts in quantum theory such as reality, locality and quantum entanglement not only deepen our understanding of quantum mechanics but also promote the development of quantum information technology. One of such debates, which has existed since the beginning of quantum mechanics, is whether the quantum state corresponds to a real physical state (i.e., it is ontic) or whether it merely represents an observer’s knowledge about the underlying reality (i.e., it is epistemic)^1^,^2^,^3^,^4^. A major reason for doubting the reality of the quantum state is that an epistemic interpretation of the quantum state could provide an intuitive explanation for many counterintuitive but fundamental quantum phenomena, such as the measurement postulate and wave function collapse^3^,^4^.

Recently Harrigan and Spekkens formulated a theory regarding the ontic and epistemic concepts of the quantum states^2^. Their model is based on a reasonable assumption that every quantum system possesses a real physical state denoted *λ*, which is called the ontic state. Every ontic state corresponds to a different ontic *λ*, on which the possibility of measurement outcomes depends. Under this assumption, quantum states are supposed to be distinguishable via two models. A state is said to be of the *ψ*-ontic model if distinct pure quantum states always match distinct real states. On the other hand, a state is of the *ψ*-epistemic model if distinct states may result in the same ontic *λ*. Pusey, Barrett, and Rudolph (PBR) further develop the argument for the *ψ*-ontic and *ψ*-epistemic models through a no-go theorem. The theorem states that *ψ*-epistemic model cannot reproduce the predictions of quantum theory if the preparation independence assumption is taken^3^. Patra *et al.*^4^ conclude on a similar result but with a different assumption of continuity for a single quantum system (Hardy also obtains a similar result independently^5^). Further theoretical works focusing on the no-go theorems for *ψ*-epistemic models have been presented in refs 6, 7, 8, 9, 10, 11, 12, 13, 14. Given these growing theoretical works, experimental tests of the *ψ*-ontic and *ψ*-epistemic models are needed.

The first experiment testing of the existence of *ψ*-epistemic models was implemented by Nigg *et al.*^15^ with two atoms in an ion trap following the PBR theorem^3^. Very recently, Ringbauer *et al.* reported an experiment to rule out the maximal *ψ*-epistemic model with single photons in 3 and 4 dimensions^16^. The no-go theory of continuous *ψ*-epistemic models^4^ was also experimentally tested using attenuated coherent states of light to simulate the high-dimensional single photon quantum states by Patra *et al.*^17^. However, due to the nonideal state preparation (the phase fluctuations of the coherent states) in the experiment^17^, one additional assumption that the ontic state depends on control measurements should be included in the explanation of the experiment. Furthermore, the no-go theorem is proposed for a state of single particles whereas a coherent state with pretty large possibility of multi-photons was used in the experiment^17^. Therefore, further experiments to test the epistemic models with a state of single particles and with fewer assumptions are needed.

In this paper, we report an experimental test of the existence of the continuous *ψ*-epistemic models with high-dimensional single photon quantum states. We produce the heralded narrowband single photon quantum states in dimensions 3, 7, 16, 25 and 32 to test the no-go theorem and our results support the no-go theorem for continuous *ψ*-epistemic models. In comparison with Patra *et al.*’s experiment^17^, our experiment benefits from the ideal single photon source and avoids the nonideal state preparation loophole. Thus the no-go theory of continuous *ψ*-epistemic models is tested without additional assumptions except for the fair-sampling assumption for the single photon detection loophole made in all of the above experiments^15^,^16^,^17^ and often made in nonlocality experiments^18^.

## Results

### The no-go theorem and the experimental setup

**Figure 1:**
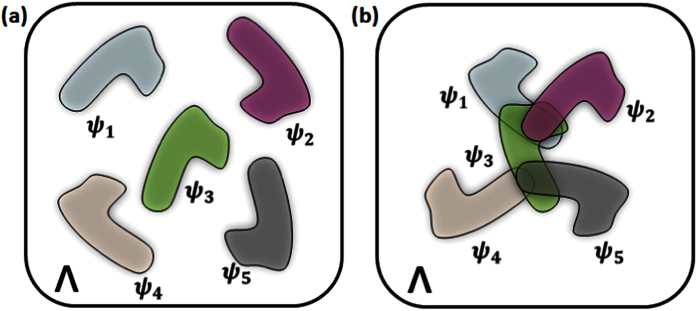
Schematic depiction of the *ψ*-ontic and *δ*-continuous *ψ*-epistemic models. Λ is the space of ontic states *λ*. (**a**) *ψ*-ontic models: distinct quantum states never overlap with each other. (**b**) *ψ*-epistemic models: different quantum states may result in the same ontic state *λ*; *δ*-continuous *ψ*-epistemic models: all states in the ball of radius *δ* share at least one ontic state *λ*, such as *ψ*_1_, *ψ*_2_, *ψ*_3_ and *ψ*_3_, *ψ*_4_, *ψ*_5_.

The difference between the *ψ*-ontic models and the continuous *ψ*-epistemic models (also named *δ*-continuous *ψ*-epistemic models) is shown in Fig. 1. The *δ*-continuous *ψ*-epistemic models assume that there are ontic states *λ* in the initial ensemble (e.g. *ψ*_1_) that will remain part of the slightly perturbed ensemble (e.g. *ψ*_2_) (see Method for a detail definition of the *δ*-continuity). There are two parameters to characterize the *δ*-continuous *ψ*-epistemic models: the parameter *δ* characterizes how continuous the model is, and 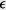 characterizes how epistemic it is. As proven in ref. 4, there is a fundamental constraint (the no-go theorem) on *δ*-continuous models for single systems: there are no *δ*-continuous *ψ*-epistemic models with 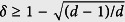 reproducing the measurement statistics of quantum states in a Hilbert space of dimension *d*. Mathematically, the *δ*-continuous *ψ*-epistemic models predict the following (with preparations *Q*_*k*_ corresponding to distinct quantum states |*ψ*_*k*_〉 all contained in a ball of radius *δ*):


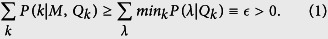


Here *P*(*k*|*M*, *Q*_*k*_) stands for the probability of outcome *k* with a measurement *M* carried out on a system in ontic state *λ* which is prepared with procedure *Q*_*k*_ and associated with a probability distribution *P*(*λ*|*Q*_*k*_). The parameter 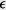 can be viewed as a measure of the extent to which distributions over real states overlap in the neighborhood of a given quantum state^4^, or it can be seen as related to the variational distance between the distributions *P*(*λ*|*Q*_*k*_)^3^.

According to quantum theory, however, there invariably exist some states for which the left-hand side of Eq. (1) is equal to 0. As a typical example, we consider *d* distinct states 
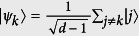
 (*j*, *k* = 1, …*d*), which are all at mutual distance 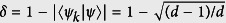 from the state 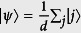. They have *δ*-continuity property since |*ψ*_*k*_〉 contain in a ball of radius *δ* centered on |*ψ*〉. If the basis of the measurement *M* is chosen to be | *j*〉, then *P*(*k*|*M*, *Q*_*k*_) = 0 for all *k* = 1, …*d* and thus the left hand side of Eq. (1) is equal to 0. Hence, the *δ*-continuous *ψ*-epistemic models’ existence can be experimentally tested.

**Figure 2:**
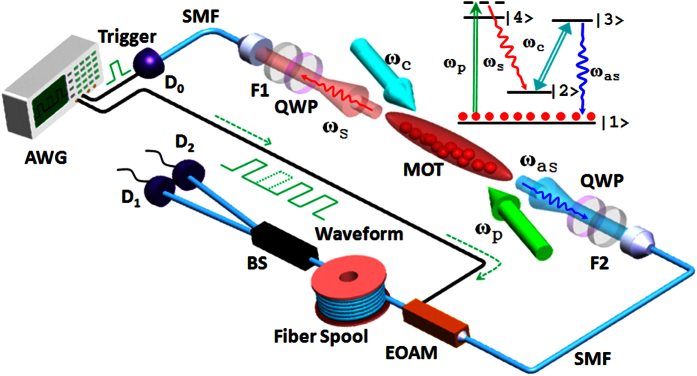
Experimental setup for producing a narrowband heralded single-photon source and testing the no-go theory for continuous *ψ*-epistemic models. The ^85^Rb atomic energy levels are chosen as 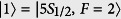, 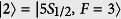, 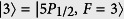, and 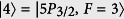. Conditioned upon the detection (*D*_0_) of the Stokes photon, its paired anti-Stokes photons pass through an EOAM driven by a train signal of *d* pulses with one missing. A beam splitter (BS) and two detectors (*D*_1_ and *D*_2_) are used to verify the single photon quantum nature.

We use the time bins of the narrowband single photon (labeled by the time *jτ* when it is detected) as the basis states | *j*〉 to realize the quantum state |*ψ*_*k*_〉 in the experiment. As shown in Fig. 2, narrowband paired photons are generated through spontaneous four-wave mixing (SFWM) in a two-dimensional ^85^Rb magneto-optical trap (MOT) (see Methods for detail)^19^,^20^. A detection (the single photon counter: PerkinElmer SPCM-AQRH, *C*_*dk*_ = 500 ± 100 *c*/*s*, *D*_0_, *D*_1_/*D*_2_) of the Stokes photon heralds the presence of its paired anti-Stokes photon, which also determines the start point of the experiment. After collection of the photons with single mode fibre (SMF), the anti-Stokes photon passes through an electro-optical amplitude modulator (EOAM, fiber-based, 10 GHz, Eospace), which is used to shape the waveform of the photons^21^,^22^. With the EOAM and an arbitrary waveform generator (AWG, Agilent 81150A), the anti-Stokes photon can be prepared with the waveform consisting of a train of *d* pulses with one missing. To perform under a stable time base, the AWG’s master clock is phase-locked to an external 10 MHz reference (SRS FS725) with exceptionally low phase noise (−130 dBc/Hz at 10 Hz offset) and one second Allan variance (<2 × 10^−11^). The modulated anti-Stokes photons then pass through a 180 *m* fiber spool to end the preparation process, which is long enough to store the entire photon wave package. The coincidence counts are implemented by a time-to-digital converter (Fast Comtec P7888) with a 2 ns bin width. The verification of the single-photon nature of the heralded anti-Stokes photons is carried out with standard Hanbury-Brown-Twiss interferometer (a fiber-based 50/50 beam splitter and two detectors *D*_1_ and *D*_2_)^23^.

### The high-dimensional single photon quantum states

**Figure 3:**
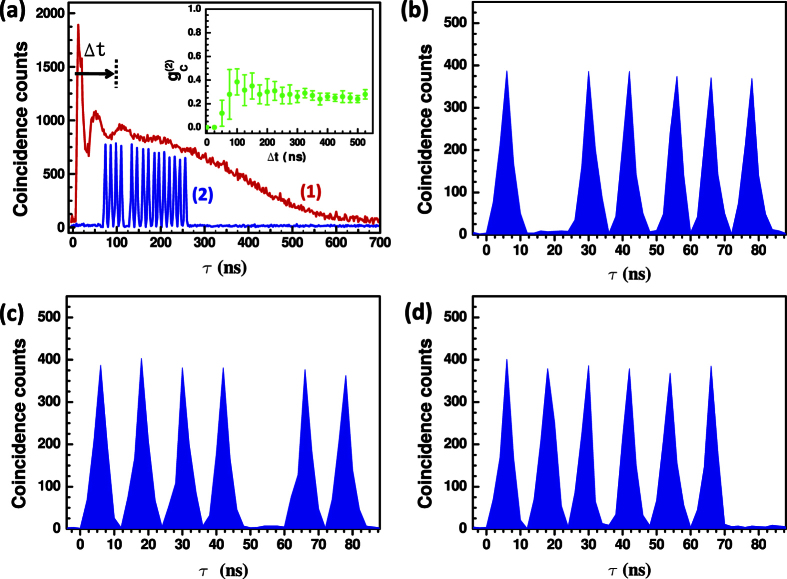
Temporal waveforms of heralded anti-Stokes photons measured as biphoton coincidence counts. (**a**) Plot (1) shows the waveform without modulation. Plot (2) is the modulated waveform of a train of pulses. Green dots (insert) represent 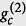 of the unmodulated anti-Stokes photons as a function of coincidence window width. Modulation pattern for states |*ψ*_2_〉 (**b**), |*ψ*_5_〉 (**c**), |*ψ*_7_〉 (**d**), in the dimension *d* = 7.

We set the pump and coupling light powers to 40 *μW* and 1.4 *mW*, respectively. With the EOAM working at its maximum transmission, the coincidence counts of the unmodulated photon pairs for 240 s are illustrated as the red curve in Fig. 3(a). The heralded anti-Stokes photon has a coherence time of about 600 ns. Controlling the EOAM with the desired square waveform allows the single photon to be modulated into a train of *d* pulses with one missing. For instance, *d* = 16 is illustrated as the blue curve in Fig. 3(a). The full width at half maximum of each pulse is about 5 ns and the time interval between neighbour pulses is 12 ns, which are limited by the bandwidth (240 MHz) of the AWG (the square waveform has a 2.5 ns rising/falling time). To minimize the defects in the rising and falling regimes of the pulse, we keep the single-photon counts only at the middle of each pulse within the time window *T*_*p*_ = 2 *ns*. The extinction ratio of the EOAM is estimated to be *R*_*ext*_ = 20 ± 2 *dB*. We confirm the quality of the heralded single-photon source by measuring its conditional autocorrelation function 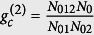, where *N*_0_ denotes the Stokes counts at *D*_0_, *N*_01_ (*N*_02_) denote the twofold coincidence counts at two detectors *D*_0_ and *D*_1_ (*D*_0_ and *D*_2_), and *N*_012_ is the threefold coincidence counts among three detectors *D*_0_, *D*_1_ and *D*_2_. An attenuated coherent light source has 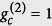, while a two-photon source gives 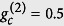. As shown in Fig. 3(a), 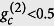 holds well within the overall waveform and it suggests the good quality of our single-photon source. Compared with the attenuated coherent light source^17^, the single-photon source reduces the multi-photon probability at least by a factor of 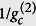 with the same generation rate.

With all the losses accounted for (the fiber coupling efficiency 70%, filter transmission 70%, EOAM transmissions 55%, detector quantum efficiency 50% and fiber connection efficiency 90%), the efficiency of our heralded narrowband single photon source is about 4.8%. In the *d*-pulse modulation case, the utilization efficiency defined as the ratio of modulated photon counts to the unmodulated photon counts, is about (*d* − 1) × 1.4%. Here 1.4% is the ratio of the counts for one pulse to the entire unmodulated photon counts. Therefore, the overall possibility of detecting one heralded anti-Stokes photon in *d*-pulse train is 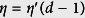 with the prefactor *η*′ = 1.4% × 4.8%. Therefore, this preparation yields the heralded single photon state as


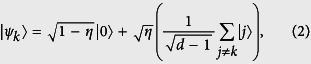


where |0〉 denotes the vacuum state, and | *j*〉 represents the single photon at time bin *jτ*.

To deal with the vacuum component that never gives rise to a click, we consider its complementary set of ontic states Λ_*clk*_ = Λ\Λ_0_ (here, Λ_*clk*_ and Λ_0_ stand for the ontic states that give rise to a click with positive probability and states that give rise to no-click, respectively)^17^. The distance should thus be redefined as 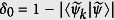, where 
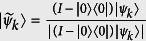
 is the projection of |*ψ*_*k*_〉 onto the space orthogonal to the vacuum, and 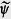 is similarly defined. Since 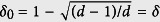 in our experiment, the above no-go theorem holds as Eq. (1) with a slight modification: for all choices of *Q*_*k*_, the *δ*_0_-continuous *ψ*-epistemic models have


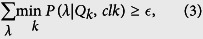


where *clk* is the event that the detector clicks in one of the time bins. Hence, the epistemic models would predict a nonzero count rate in the unregistered bins which contradicts the quantum prediction. In addition, we would like to point out that the *δ*_0_-continuous *ψ*-epistemic models will still predict a nonzero counts as shown in Eq. (3) even in the presence of inefficient detectors under the fair-sampling assumption^17^,^24^. This class of *ψ*-epistemic models are what we test in the experiment.

### Experimental results

**Figure 4:**
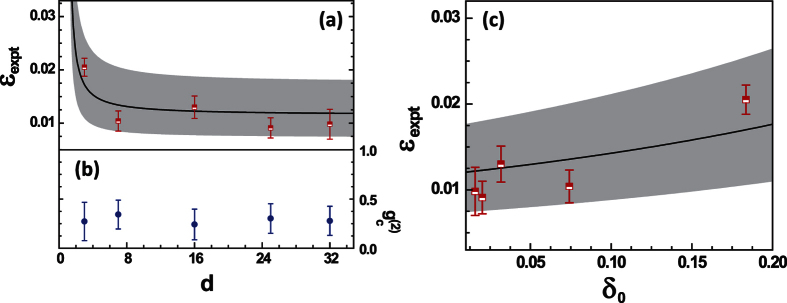
(**a**) Experimental boundary on the *ψ*-epistemic models: the measured value of 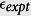 (open red squares; left axis) as a function of the dimension *d*. The solid black line is the 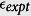 estimated by Eq. (6). (**b**) The conditional correlation function 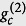 (solid blue circles; right axis) of the heralded single photons. (**c**) The dependency of the measured value 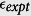 (open red squares) on the distance *δ*_0_. The solid line is the 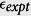 calculated by Eq. (6) with the substitution 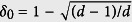. The gray zones in (**a**,**c**) are the areas in which the quantum theory prediction could vary, taking into account the uncertainty on dark-count rate *C*_*dk*_ = 500 ± 100 *c*/*s*, extinction ratio *R*_*ext*_ = 20 ± 2 *dB*, and overall detection probability *η* = (0.07 ± 0.003)%(*d* − 1). Error bars represent the statistical errors.

In our experiment, we produce the state *ψ*_*k*_ (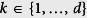) for several dimensions *d* = 3, 7, 16, 25, 32. As an example, the measured results for |*ψ*_2_〉, |*ψ*_5_〉, and |*ψ*_7_〉 at *d* = 7 are shown in Fig. 3. The measured fraction of counts in the unregistered time bins 
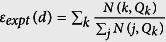
 (where *N*(*j*, *Q*_*k*_) represents the counts registered in bin *j* when we prepare state |*ψ*_*k*_〉) and their statistical errors for different dimensions *d* are shown in Fig. 4 (open red squares and red bars). As expected, the count rate in the empty bins is not strictly zero due to the imperfect optical components. The expected quantity *ε*_*expt*_ can be obtained through the estimation from the finite extinction ratio *R*_*ext*_ of the EOAM and the detector dark counts *C*_*dk*_*T*_*p*_ during a time bin, i.e.,


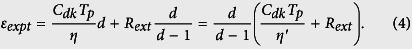


The dependency of 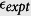 on *d* calculated by Eq. (4) is plotted in Fig. 4(a) (black line). In addition, the uncertainty of 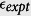 are shown in the gray region. Any positive deviation from this gray region could support an epistemic model. The fact that the measured values of *ε*_*expt*_ reveal no positive deviation excludes a large class of *ψ*-epistemic models.

Since the quality of the single-photon source is crucial, we also measure the conditional autocorrelation function 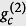 for each dimensions *d* and the results are shown in Fig. 4(b). It shows that the measured 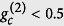 is kept within the whole coincidence window of the modulated anti-Stokes photon. Furthermore, in order to shown which kinds of continue *ψ*-epistemic models are excluded in our experiment, we plot the experimental bound in the *δ*_0_, 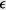 plane in Fig. 4(c). As shown in Fig. 4(c), the models with 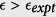 are ruled out in the experiment.

## Discussions and Conclusion

Our experiment is based on the theoretical work of ref. 4. Comparing with the previous experiment of ref. 17, our experiment utilizes a heralded narrowband single photon source, which has several merits that advance the test of the no-go theory of continuous *ψ*-epistemic models. First, the state we use is a really pure quantum state rather than an equivalent pure state obtained by weakening a laser beam. Second, in our experiment, the nonideal state preparation loophole is plugged up for the utilization of the single photon Fock state that is immune to phase fluctuation^25^,^26^. This greatly simplifies the modification of the tested *ψ*-epistemic models and also avoids the need to introduce additional assumptions. Furthermore, the overall efficiency *η* in Eq. (6) is not constant but depends on *d*, which leads to two significantly new results when compared with ref. 17. First, the *δ*-continuous *ψ*-epistemic models are characterized by two parameters *δ* and 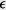. Logically, when distinctive quantum states become closer to each other, they should share more common ontic states *λ* in the ontic space Λ. In other words, *δ*-continuous *ψ*-epistemic models allow the parameter 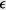 to increas when the distance *δ* decreases. However, as shown in Fig. 4(c), the experimentally measured quantity 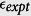 clearly decreases when the distance *δ*_0_ decreases, which is counterintuitive in the view of the epistemic models. Second, as shown in Fig. 4(a), the experimentally measured quantity 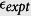 decreases when the dimension *d* increases. Therefore if we use a better single photon detector with fewer dark counts (e.g., a superconducting single-photon detector) and a better modulator with higher extinction ratio to reduce the undesired counts, the measured quantity 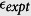 can possibly be arbitrarily small. It implies that, for any given *δ*_0_ and 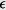, one can always find an appropriate dimension *d* and detecting apparatus so that 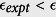, which suggests a reliable method to exclude almost all kinds of continuous *ψ*-epistemic models.

In summary, we have reported an experiment to test the no-go theorem for *δ*-continuous *ψ*-epistemic models with high-dimensional single photon quantum states. The tested no-go theorem is based on a natural assumption of continuity. However, as proposed in ref. 27, the *δ*-continuity does not hold well for all preparation procedures, one of such example is the purely classical distributions of the form 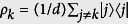. So there are debates^27^ on the validity of the no-go theorem in ref. 4. Nevertheless, the continuity may hold for those associated with pure quantum states. Here, the heralded single photon has a purity of *γ* > 0.98 (see Methods for detail analysis). Hence, the assumption of continuity is reasonable in our experiment. Last, we also would like to point out that there still exist some *ψ*-epistemic models which are not *δ*-continuous, such as pairwise continuous *ψ*-epistemic models^11^. Thus, whether a no-go theory exists for all the *ψ*-epistemic models is still an open question deserving further exploration.

## Methods

### Definition of the *δ*-continuity

Let *δ* > 0 and let 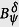 be the ball of radius *δ* centered on |*ψ*〉, i.e., 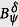 is the set of states |*ϕ*〉 such that 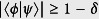. If for any preparation *Q*, there exists an ontic state *λ* (which can depend on *Q*) for all preparations *Q*′ corresponding to quantum states 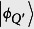 in the ball 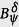 centered on the state |*ϕ*_*Q*_〉, we have *P*(*λ*|*Q*′) > 0. Then we say this model is *δ* continuous.

### The narrowband paired photon source

The narrowband photon pairs are generated through spontaneous four-wave mixing (SFWM) in a two-dimensional ^85^Rb magneto-optical trap (MOT) with a longitudinal length of *L* = 1.7 *cm* as addressed in our previous work^20^,^28^. The atoms are prepared on the ground level |1〉 and have an optical depth of about 50 in the |1〉 → |3〉 transition, as shown in Fig. 2. The pump laser (780 nm, *ω*_*p*_) is 80 MHz blue detuned from the transition |1〉 → |4〉, and the coupling laser (795 nm, *ω*_*c*_) is on resonance with the transition |2〉 → |3〉. The Stokes and anti-Stokes beams, focused at the MOT center with a 1/*e*^2^ diameter of 0.3 mm, are aligned at a 2.5° angle with respect to the pump-coupling axis. In the presence of two counter propagating pump and coupling lasers, phase-matched Stokes (*ω*_*s*_) and anti-Stokes (*ω*_*as*_) photon pairs can be generated. After the quarter wave plates (QWP) and filters (F1/F2), the photon pairs are collected by single-mode fibers (SMF) and finally detected with single-photon counters (PerkinElmer SPCM-AQRH, *C*_*dk*_ = 500 ± 100 *c*/*s*, *D*_0_, *D*_1_/*D*_2_). The source is run periodically with a magneto-optical trap for a trapping time of 4.5 ms and a biphoton generation time of 0.5 ms.

### The purity of the heralded single photon

The narrowband time-frequency entangled paired photons generated through SFWM are used to produce the heralded single photon. The time-frequency quantum-state purity of the heralded single photon depends on the response time uncertainty of the trigger photon detector^25^: 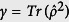, where 
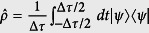
 is the density operator of the heralded single photon, and Δ*τ* is the response time of the detector. With a typical time resolution of Δ*τ* = 2 *ns* and the power spectrum bandwidth of the source being smaller than 5 *MHz*, the heralded single photon has a purity of *γ* > 0.98.

## Additional Information

**How to cite this article**: Liao, K.-Y. *et al.* Experimental test of the no-go theorem for continuous **ψ**-epistemic models. *Sci. Rep.* **6**, 26519; doi: 10.1038/srep26519 (2016).
